# Supplementary figures and images for: Cycloheximide-Producing Streptomyces Associated With Xyleborinus saxesenii and Xyleborus affinis Fungus-Farming Ambrosia Beetles
Source: Front Microbiol. 2020 Sep 24;11:562140. doi: 10.3389/fmicb.2020.562140 (PMC7546818; doi:10.3389/fmicb.2020.562140)

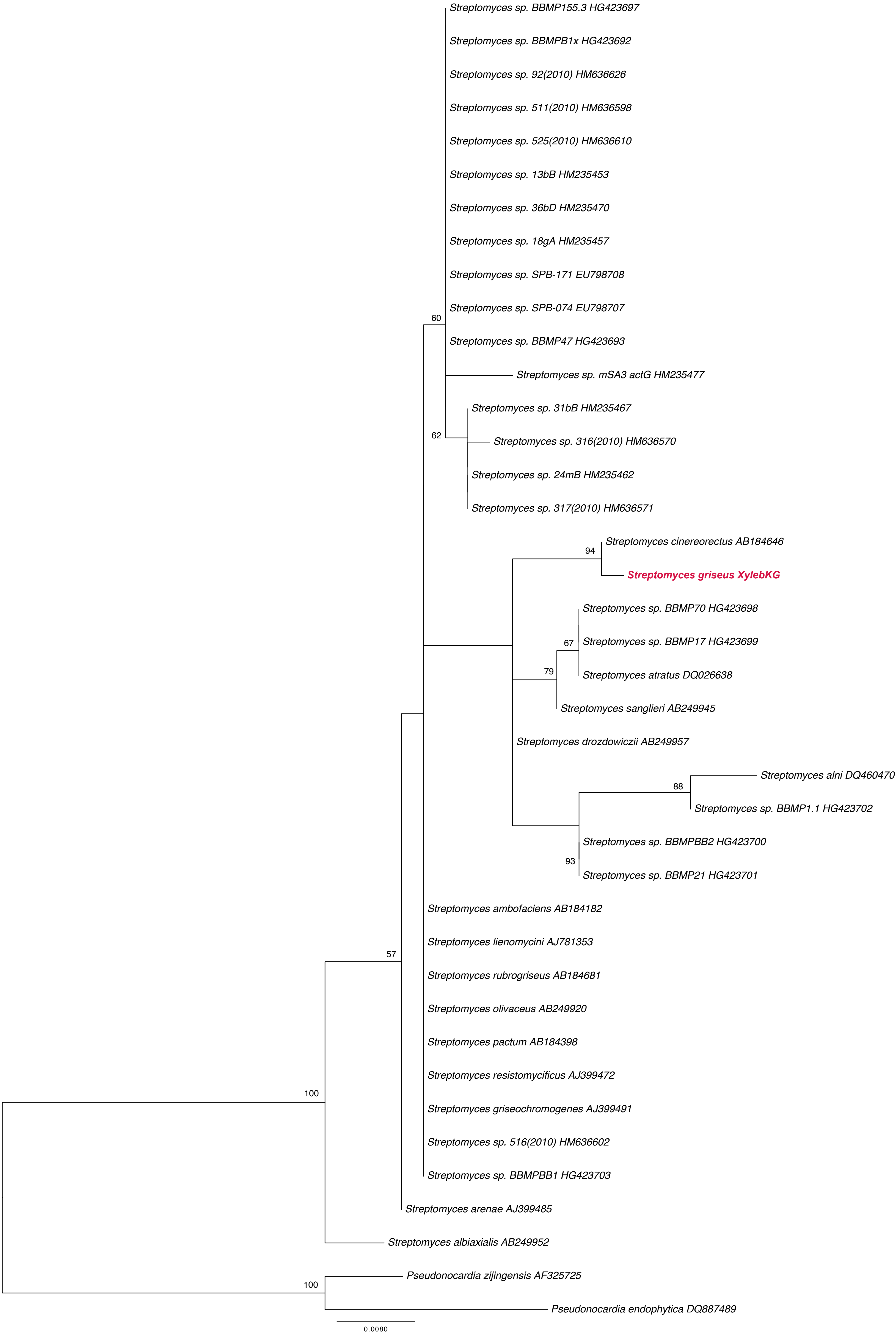

Supplement: Supplementary Figure 1 — Updated Maximum likelihood 16S phylogeny of the XylebKG-1 clade and its relatives. [file Data_Sheet_2.PDF]

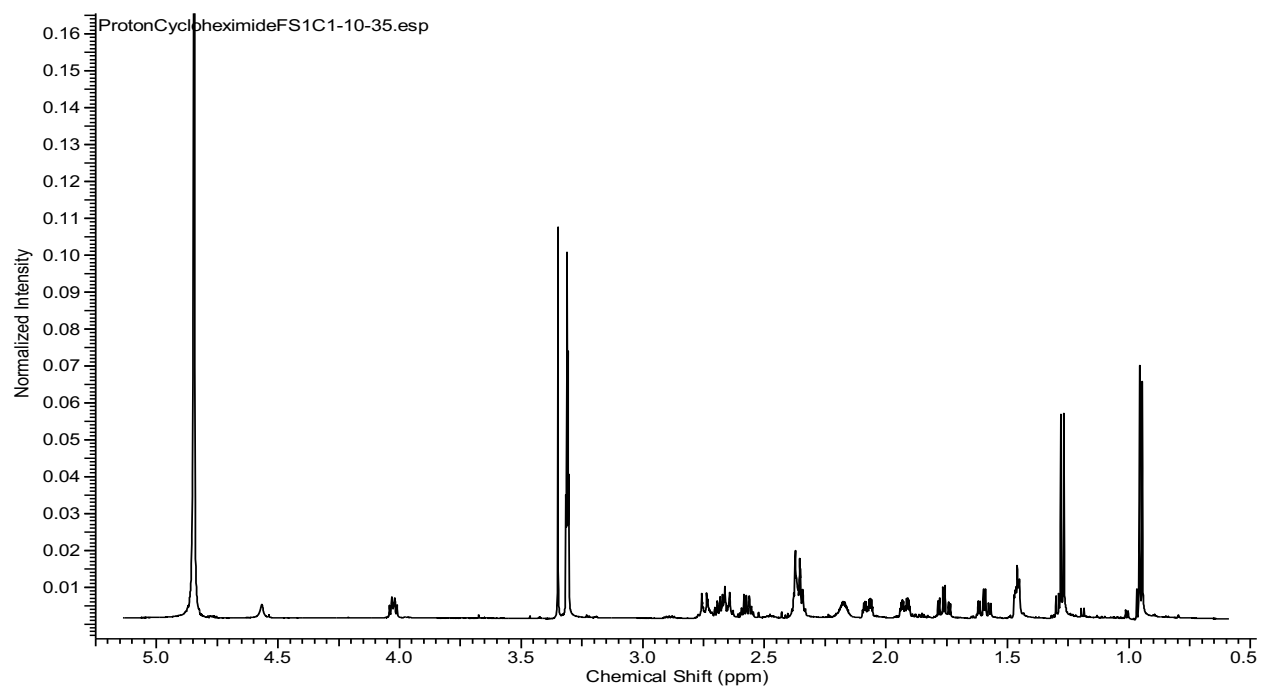

Supplement: Supplementary Figure 2 — 1H spectrum of cycloheximide (CD3OD). [file Data_Sheet_3.PDF]

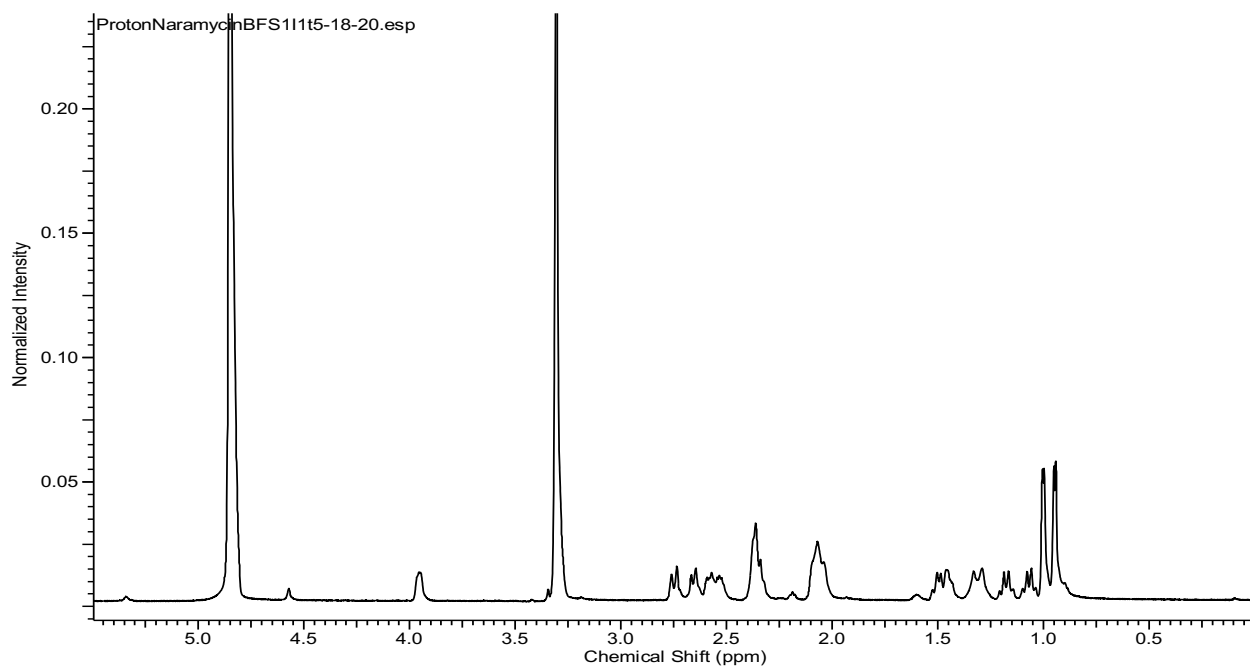

Supplement: Supplementary Figure 3 — 1H spectrum of naramycin B (CD3OD). [file Data_Sheet_4.PDF]

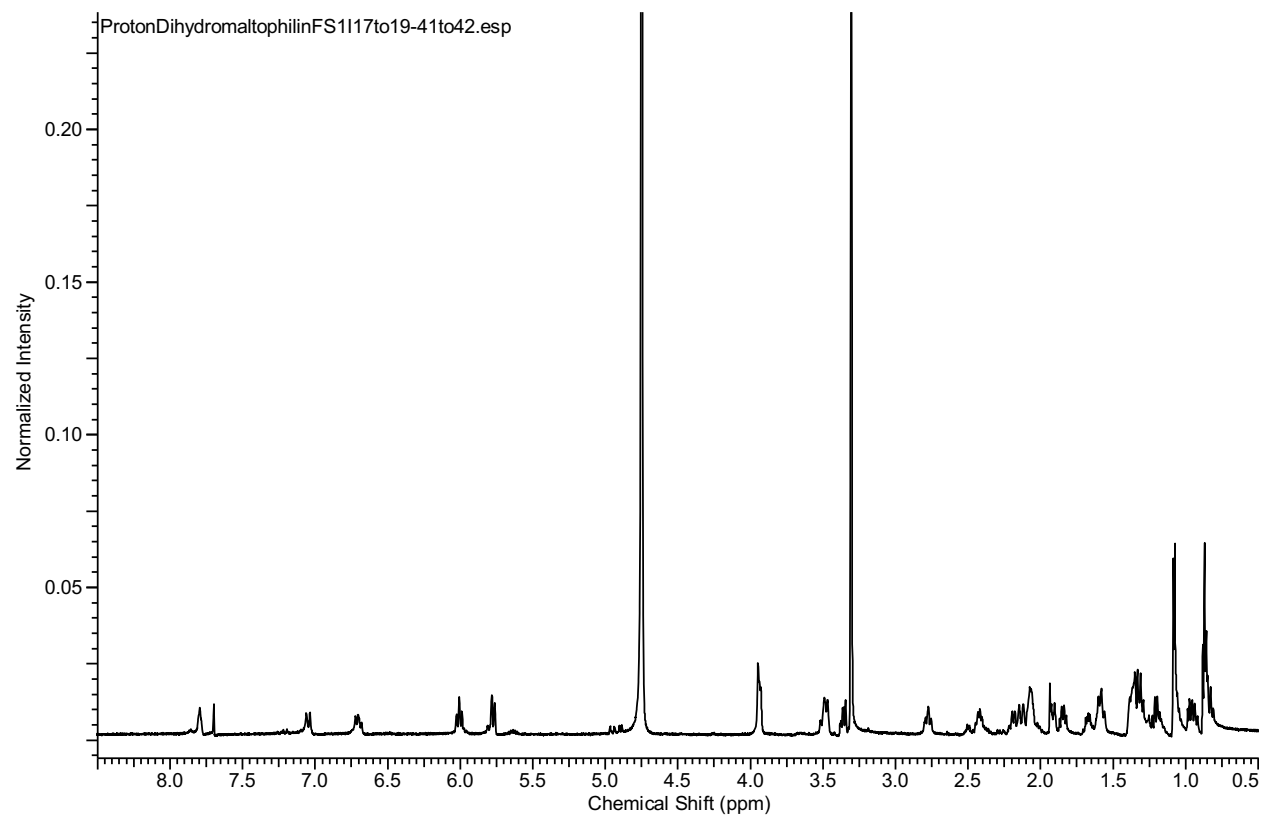

Supplement: Supplementary Figure 4 — 1H spectrum of dihydromaltophilin (CD3OD). [file Data_Sheet_5.PDF]

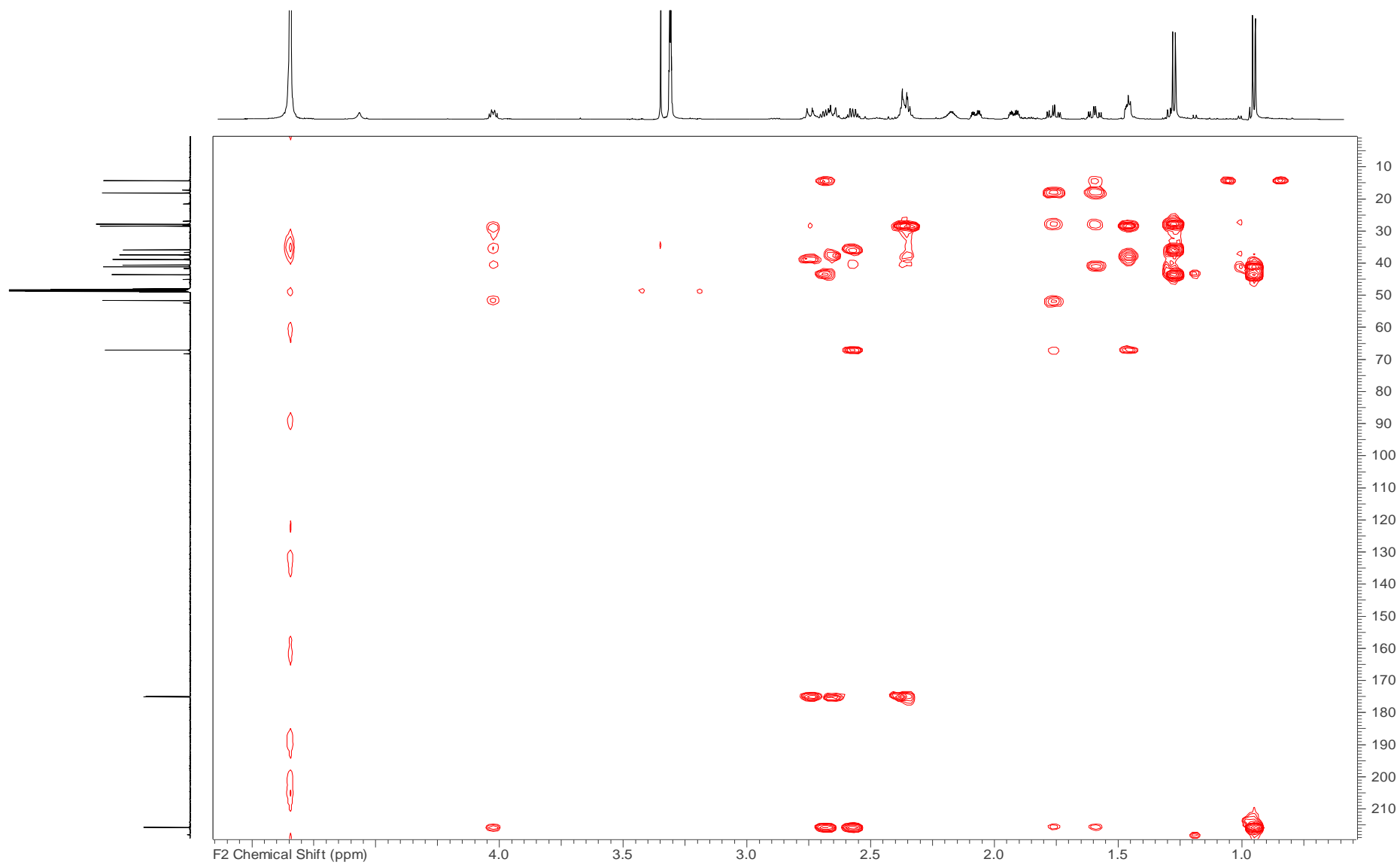

Figure S6: HMBC spectrum (600 MHz, methanol- $d_4$ ) of cycloheximide (**1**).

Supplement: Supplementary Figure 5 — HMBC spectrum (600 MHz, methanol-d4) of cycloheximide (1). [file Data_Sheet_6.PDF]
